# Integrated Models of Care for Individuals with Opioid Use Disorder: How Do We Prevent HIV and HCV?

**DOI:** 10.1007/s11904-018-0396-x

**Published:** 2018-05-17

**Authors:** Katherine M. Rich, Joshua Bia, Frederick L. Altice, Judith Feinberg

**Affiliations:** 1 Section of Infectious Diseases, AIDS Program, Yale School of Medicine, New Haven, CT USA; 2 Frank H. Netter School of Medicine, Quinnipiac University, North Haven, CT USA; 3 Centre of Excellence on Research in AIDS (CERIA), University of Malaya, Kuala Lumpur, Malaysia; 4 Departments of Behavioral Medicine & Psychiatry and Medicine, West Virginia University School of Medicine, Morgantown, WV USA

**Keywords:** Opioid use disorder, Hepatitis C virus, HIV, Integrated care, Co-located care

## Abstract

**Purpose of Review:**

To describe models of integrated and co-located care for opioid use disorder (OUD), hepatitis C (HCV), and HIV.

**Recent Findings:**

The design and scale-up of multidisciplinary care models that engage, retain, and treat individuals with HIV, HCV, and OUD are critical to preventing continued spread of HIV and HCV. We identified 17 models within primary care (*N* = 3), HIV specialty care (*N* = 5), opioid treatment programs (*N* = 6), transitional clinics (*N* = 2), and community-based harm reduction programs (*N* = 1), as well as two emerging models.

**Summary:**

Key components of such models are the provision of (1) medication-assisted treatment for OUD, (2) HIV and HCV treatment, (3) HIV pre-exposure prophylaxis, and (4) behavioral health services. Research is needed to understand differences in effectiveness between co-located and fully integrated care, combat the deleterious racial and ethnic legacies of the “War on Drugs,” and inform the delivery of psychiatric care. Increased access to harm reduction services is crucial.

**Electronic supplementary material:**

The online version of this article (10.1007/s11904-018-0396-x) contains supplementary material, which is available to authorized users.

## Introduction

The opioid epidemic in the USA, especially the rise in injection drug use (IDU), has ushered in a volatile and evolving risk environment for HIV and hepatitis C (HCV) [1–5], particularly in rural areas [2, 6]. In 2016, over 2 million Americans had an opioid use disorder (OUD), with 11.5 million people aged 12 years or older misusing prescription painkillers and 948,000 using heroin and/or synthetic opioids such as fentanyl [7]. Currently, 10–20% of people who misuse prescription opioids escalate to injection [8, 9], contributing to substantial risk for new HIV and HCV outbreaks due to the sharing of contaminated injection equipment and sexual risk behaviors [10].

This HIV/HCV risk environment is epitomized by the outbreak in Scott County, Indiana beginning in late 2014 [11]. HIV incidence, attributed to shared injection equipment, rose from 5 to 181 cases in 2015. Over 90% were co-infected with HCV. Nationally, 2392 new HIV cases in people who inject drugs (PWID) were reported in 2015 [3]. HCV incidence tripled between 2010 and 2015, primarily due to opioid-related drug injection [12]. HIV and HCV prevention efforts for individuals with OUD have been challenged by lack of health insurance [13], limited access to medication-assisted treatment (MAT) [14, 15], fragmented care [16], psychiatric co-morbidities [3], and the syndemic nature of these overlapping epidemics where services that are most needed do not exist [16, 17].

The design and scale-up of integrated or co-located, multidisciplinary care models that engage and retain individuals is critical to the success of HIV and HCV prevention strategies [16, 18, 19]. Key components of such models are (1) provision of MAT to reduce risks for acquiring HIV and HCV and to prevent HCV re-infection, (2) HIV and HCV treatment to prevent transmission within injection drug and sexual networks (“treatment as prevention, TasP”), (3) access to HIV pre-exposure prophylaxis (PrEP), (4) behavioral health services that address psychiatric co-morbidities and increase engagement with and adherence to care, and (5) access to harm reduction services (i.e., sterile injection equipment and naloxone) [16, 18–22]. This “twenty-first century” opioid epidemic, initially demographically and geographically different from the “twentieth century” one, is characterized by PWID networks among rural, primarily white populations [2, 23, 24], although it has recently extended to urban minority communities [25••]. This means that the delivery of integrated or co-located care will need to be tailored to different populations, geographical locations, and the surrounding, typically resource-poor, healthcare environments [26••].

Understanding models of care that have integrated or co-located HIV, HCV, and OUD services is crucial for optimizing initiatives to expand access to care. This paper presents current models of care within five treatment settings: primary care, HIV care, specialized opioid treatment programs (OTPs), transitional clinics, and community-based harm reduction programs. Program characteristics, as well as a representative model of care for each treatment setting, are described to guide future program development, followed by a discussion of emerging approaches and areas that need further research.

## Methods

### Search Strategy

We searched the literature describing care models that included MAT (methadone, buprenorphine, naltrexone), HIV, and/or HCV services from August 2007 to September 2017 using Ovid Medline, Scopus, PsychInfo, and EMBASE databases for articles and conference abstracts (see Appendix Table S.1 for search terms). We also reviewed reference lists of key papers describing integrated/co-located models of care. To be included, studies had to describe a health service(s) delivery program that (1) was located in the USA, (2) provided MAT, (3) provided HIV and/or HCV care, (4) was designed for adults (≥ 18 years), and (5) was written in English or Spanish.

### Data Extraction

Selected studies were classified as describing care provided in one of the five treatment settings listed above. For each model, data were extracted to describe the population(s) served, the OUD treatment agents available, and the extent or co-location of mental healthcare integration and HIV/HCV care.

### Selection of Representative Model

For each treatment setting, we selected a representative model of care based on their similarity to other programs within the given category, their innovativeness, or their focus on HIV and/or HCV care.

## Results

We identified 1394 abstracts and reviewed 141 full text articles and conference abstracts, of which 41 described 17 unique, co-located or integrated models of care (5 HIV specialty care, 3 primary care, 6 OTP, 2 transitional clinics, and 1 community-based harm reduction program), and two emerging models. Details of each of model of care are provided in the Appendix (Table S.2). General characteristics of the populations served by treatment setting are provided in Table 1. Pharmacological agents used to treat OUD are described in Table 2.

**Table 1:** Characteristic of care models by treatment setting

Treatment setting	Populations served	Available MAT
Office-based primary care	All individuals who have access to a PCP	Buprenorphine, naltrexone
HIV care sites	1. Urban populations and/or populations with a high prevalence of HIV 2. HIV-positive individuals who are linked to treatment	Buprenorphine, naltrexone
Specialty opioid treatment programs	1. Urban populations 2. Individuals with an OUD who are linked to treatment	Methadone, buprenorphine, naltrexone
Community-base harm reduction programs	1. Communities with a high prevalence of PWID 2. PWID and individuals without linkage to other health care services	Buprenorphine, naltrexone
Transitional clinics	1. Communities with high rates of incarceration	Buprenorphine, naltrexone

**Table 2:** Characteristics of pharmacologic agents for OUD treatment

Agent	Mechanism	Dosing schedule	Location availability	Regulations
Methadone	μ receptor full agonist	Daily	OTP	Methadone must be provided at a federally regulated specialty OTP
Buprenorphine (buccal or sublingual)	μ receptor partial agonist (often paired with antagonist)	Daily	Office-based, OTP, harm-reduction programs, HIV care sites	Physicians must complete 8-h training and receive a DEA waiver Nurse practitioners and physician assistants must complete 24 h of training and received a DEA waiver
Buprenorphine (implantable)	μ receptor partial agonist	Once every 6 months	Office-based, OTP, HIV care sites	Physicians must have waiver (8-h training required)
Naltrexone (oral)	μ receptor antagonist	Daily	Office-based, OTP, HIV care sites	Any provider who can prescribe medications
Naltrexone (injection)	μ receptor antagonist	Once per month	Office-based, OTP, HIV care sites	Any provider who can prescribe medications

### Primary Care

A key strategy to expand access to comprehensive care for individuals with OUD, and thereby decrease the risk of HIV and HCV transmission, is the integration or co-location of treatment for all three conditions into primary care settings [26••], particularly in rural areas that lack specialty opioid and infectious diseases treatment centers. Key advantages of the delivery of OUD, HIV, and HCV treatment in the primary care setting are that this model lowers the threshold for patient entry and enhances continuity of care [27, 28]. As many individuals with opioid misuse or addiction seek treatment for physical ailments, primary care providers (PCPs) play a crucial role in identifying substance use disorders (SUDs) and expanding treatment accessibility [29, 30]. Physicians who complete 8 h of training and nurse practitioners and physician assistants who complete 24 h of training may obtain a Drug Enforcement Administration (DEA) waiver to prescribe buprenorphine or buprenorphine/naloxone, the mainstay of MAT [31, 32]. In addition to treating OUD, buprenorphine/naloxone also has been shown to reduce HCV reinfection [33]. For individuals with OUD who do not want buprenorphine, any clinician can prescribe extended-release naltrexone (XR-NTX), an evidence-based opioid antagonist treatment for OUD that requires successful completion of supervised withdrawal (“detox”) before starting treatment.

#### Representative Example:

##### Location

A federally qualified health center (FQHC) affiliated with Montefiore Medical Center in the Bronx, New York, that serves over 9000 adults annually [34•].

##### MAT Services

A team of internists and clinical pharmacists conduct screening and onsite buprenorphine/naloxone treatment, including induction, stabilization, and maintenance. Clinical pharmacists, internists, or both together conduct follow-up visits. Motivational interviewing is incorporated into medical visits to promote adherence and engagement in care. Clinical judgment drives urine drug testing (UDT), but no one is discharged from treatment solely due to UDT results [34•, 35•].

##### Psychiatric Services

Co-located.

##### HIV Services

Testing and treatment are provided, supported by Ryan White Care Act funding [34•, 35•].

##### HCV Services

HCV evaluation and treatment are provided by a physician trained in both HCV care and addiction medicine, supported by a care coordinator. Referrals come from PCPs and nearby syringe exchange programs. Treated patients are enrolled in the NYC Department of Health’s Check Hep C Patient Care Coordinator Program which provides a full time care coordinator to facilitate linkage and retention in HCV care, psychosocial assessment, health education, navigation of insurance prior authorization and claims, and applications to patient assistance programs for medication [34•].

### HIV Specialty Care Clinics

HCV prevalence among people living with HIV/AIDS (PLWH) is about 25% nationally [36], and HIV/HCV co-infection among PWID ranges between 50 and 90% [37]; both vary by location and population served. Specialty HIV care sites, which typically provide access to infectious disease experts, are an ideal setting to provide integrated or co-located HIV, HCV, and OUD treatments in communities with a high prevalence of HIV among PWID.

Acknowledging the importance of integrating substance use treatment at HIV care sites, the HIV/AIDS Bureau of the Health Resources and Services Administration (HRSA) funded the Buprenorphine and HIV Care Evaluation and Support (BHIVES) initiative from 2004 to 2009. BHIVES developed and implemented integrated HIV care and buprenorphine/naloxone treatment at ten sites [38]. Qualitative interviews with patients at one site suggest that they felt positively about the provision of MAT at an HIV care site, as it allowed access to OUD treatment in a setting that did not care exclusively for individuals with SUDs. In a longitudinal analysis, HIV patients prescribed and maintained on buprenorphine/naloxone were significantly more likely to be prescribed antiretroviral therapy (ART) and to achieve viral suppression than those not retained on MAT [39]. Key features associated with success at each clinic involved ongoing education and support to providers [40] and a “glue” person who coordinated care for these more complex patients [38].

#### Representative Example

##### Location

A hospital-based HIV clinic in Providence, Rhode Island. The clinic cares for 1400 HIV-infected patients, of whom approximately 30% have OUD [41•].

##### MAT Services

Patients are regularly informed of onsite buprenorphine/naloxone treatment through nurse-led educational sessions and during regularly scheduled HIV care appointments. The clinic also coordinates with nearby substance use treatment facilities.

##### Psychiatric Services

Psychologists and social workers provide both individual care and support groups [42].

##### HIV Services

Specialty HIV care is provided by HIV physicians. The clinic, embedded within a hospital facility, provides HIV testing, counseling, treatment, education, and case management [42].

##### HCV Services

All patients are screened, evaluated, and provided treatment onsite through the HIV program’s co-located Coinfection Clinic [41•].

### Specialty OTPs

Opioid treatment programs (OTPs) are federally qualified programs that specialize in methadone maintenance therapy (MMT) [43]. OTPs can be funded either directly or indirectly through public sources or through fee-for-service models. An advantage of MMT in the public setting is that it is usually provided with minimal direct expense to the patient [44]. MMT requires daily visits to receive methadone; although with time and duration of abstinence, patients can take home doses for self-administration [45]. OTPs may also be staffed by other healthcare providers who can provide primary or specialty care for a range of co-morbid psychiatric conditions, HIV and HCV [46•]. HCV-infected individuals who are treated while receiving MAT have achieved high rates of sustained virologic response (SVR, or cure) irrespective of continued drug use [33], underscoring the fact that sobriety should not be a precondition of HCV treatment.

#### Representative Example

##### Location

The APT Foundation, a non-profit addiction treatment program in Connecticut, where the majority of patients have an OUD [46•]. Injection equipment disposal boxes are installed in exam rooms.

##### MAT Services

All patients undergo an initial medical and psychiatric evaluation. The program provides methadone or buprenorphine to over 5000 patients [46•].

##### Psychiatric Services

Psychiatric care, including psychotherapy, individualized treatment counseling and medication, is available [46•].

##### HIV Services

HIV testing, counseling, and treatment are provided; referrals can also be made to specialty clinics.

##### HCV Services

HCV testing, counseling, and treatment are provided. Free voluntary HCV screening is provided, and for those with infection, their contacts may be approached anonymously. There is supportive care from social workers and psychiatrists as needed [46•].

### Community-Based Harm Reduction Programs

Syringe service programs (SSPs, also known as syringe or needle exchange programs) are a critical component of HIV, HCV, and overdose prevention [47–52]. SSPs provide non-judgmental, free, accessible testing and care services to PWID who often do not have contact with medical services or who shun medical care because of stigma and prior poor treatment [53]. A recent study found that 50% SSP coverage within the USA would be cost-effective and avert up to 35,000 HIV infections over 20 years [54]. For maximal impact, SSPs must provide a robust array of health services [55–57], including HIV testing linked to both primary (e.g., sterile equipment, addiction treatment, PrEP) and secondary HIV prevention (e.g., TasP), and HCV testing with linkage to parallel services. A modeling study found that a significant reduction in HIV transmission within a community with an established epidemic requires that SSPs provide multiple services, including linkage to healthcare and MAT [55]. In parallel, a meta-analysis concluded that SSPs that provide only sterile syringes are insufficient to prevent the spread of HCV; rather, SSPs must also provide sterile injection equipment (i.e., cottons, cookers), as well as other harm reduction services (i.e., safe sex, condoms, and sterile injection education) [56]. A network model found that if HCV prevalence within a PWID community is ≤ 60%, then treating ≥ 12% of HCV-positive PWID in the network would eliminate HCV within 10 years [58]. Together, these data support the concept that the ideal SSP is a “one-stop shop” that provides harm reduction, HIV and HCV testing and linkage to services and linkage to OUD treatment services.

#### Representative Example

##### Location

A mobile medical clinic and an associated fixed site clinic in Connecticut. Bilingual (English/Spanish) case managers provide outreach services using a minivan [59•].

##### Harm Reduction Services

Sterile injection equipment, condoms, and rapid HIV and HCV testing are provided at both sites. Naloxone, overdose prevention education, and safe sex education are offered at each visit.

##### MAT Services

Maintenance with buprenorphine/naloxone or naltrexone (XR-NTX) is provided at both sites.

##### Psychiatric Services

Social workers and psychiatric nurse practitioners provide mental health counseling at the fixed site clinic.

##### HIV Services

HIV treatment and case management is provided at both sites, and ART is dispensed as directly administered therapy [59•]. There are no specialty services for complicated HIV patients; however, linkage to off-site specialty HIV care is available.

##### HCV Services

Rapid HCV tests are provided. Outreach workers provide case management services and linkage to HCV treatment.

### Transitional Clinics

Individuals transitioning from criminal justice settings (CJS) back to the community have a disproportionately high prevalence of HIV, HCV, substance use, and psychiatric illness (for review, see [60]). One in six PLWH transitions through a prison or jail annually [61]. While most PLWH achieve viral suppression within the CJS [62], linkage to and retention in care post-release are suboptimal [63], with relapse to drug and alcohol use associated with poor ART adherence and retention in care [64]. Moreover, release from prison is associated with extraordinary mortality primarily due to overdose fatality, especially within the first 2 weeks post-release.

Like HIV, people with HCV are concentrated in CJS; 11 to 37% have HCV, representing 30% of the estimated 5.7 to 6.5 million cases in the USA [65]. HCV treatment within prison has been feasible with good outcomes [66], and newer data using direct-acting antivirals (DAAs) in New York confirm earlier findings [67]. Nonetheless, in 2015, less than 1% of prisoners known to be HCV-positive were provided treatment while incarcerated, largely due to the high cost of DAA and limited funding for treatment [68]. Accordingly, many inmates with untreated HCV transition into the community, challenging linkage to care efforts and potentially fueling onward HCV transmission, undermining both the health of the individual and the community.

This evidence underscores the importance of transitional clinics specifically designed to care for individuals who have recently been released from incarceration, with the aim of optimizing care coordination between the CJS and the community. Transitional clinics can increase engagement and retention in care and will have the greatest impact in communities with high rates of incarceration. Importantly, this model may help address current racial disparities in access to HIV, HCV, and OUD care, as well as treatment outcomes among black individuals, who have the highest rate of new HIV infections and AIDS diagnoses [69].

#### Representative Example

##### Location

The Bronx Transitions Clinic (BTC) operates an “open access” clinic from an federally qualified health center (FQHC) affiliated with Montefiore Medical Center. The program is a collaboration between the clinic and the Osborne Association, a community-based organization that screens inmates for chronic health conditions and initiates case management while they are still incarcerated. The BTC provides a range of services on a sliding fee scale. Specific educational programs help facilitate community reentry. Formerly incarcerated community health workers are employed to provide health education, social support, and care coordination [70•, 71•].

##### MAT Services

All individuals with OUD are offered buprenorphine/naloxone.

##### Psychiatric Services

Provided onsite.

##### HIV Services

ART and case management are provided; approximately 20% are HIV-positive. The Osborne Association provides case management through outreach workers.

##### HCV Services

Treatment is provided onsite.

### Emerging Models

The volatile nature of the opioid epidemic, its associated risk of new HIV and HCV outbreaks, the availability of new, effective treatments, and the rural location of many PWID necessitate the development of innovative care models. Expanded access to care in rural areas is desperately needed.

In 2012, it was estimated that state-level rates of buprenorphine treatment capacity ranged from 0.7 to 13.8 patients per 1000 individuals aged 12 years or older [72]. All but two states had a rate of past year OUD greater than their treatment capacity rate, resulting in an enormous gap between treatment need and capacity of 1.4 million people [72]. A survey of 108 PCPs in 2014 found that while 80% reported that they knew of opioid-dependent patients and 73% admitted to feeling a personal responsibility as a primary care doctor to treat addiction, only 10% prescribed MAT [73]. The most commonly cited reasons for not providing MAT was the belief that treating addiction is difficult, a lack of confidence in prescribing according to guidelines, and a low level of staff preparedness. Similar barriers exist for the provision of HIV and HCV care, particularly for individuals with OUD [74–76].

A second factor contributing to the treatment gap is the changing demographic characteristics and geographic distribution of individuals with OUD [23, 77•]. Historically, opioid use was largely concentrated in urban areas among minority populations, whereas currently it is concentrated among primarily white populations in suburban and rural areas [23]. In an analysis of county-level vulnerability to HIV and HCV infections, 220 rural counties were defined as vulnerable [77•], reflecting the changing geographic distribution of both opioid misuse and addiction, and the associated increase of IDU.

There are two emerging models of care that hold promise for expanding access to integrated care services by utilizing internet and cell phone technologies: Project ECHO (a tele-education program) [78] and a A-CHESS (an mHealth phone application) [79•].

#### Telemedicine and Tele-education

Standard telemedicine via two-way videoconferencing permits direct interaction between patients and specialists in different locations. It is an adaption of the traditional brick-and-mortar “hub and spoke model”, in which a specialty care center provides support and resources to several lower-tier care centers throughout a geographic area.

Project Extension for Community Care Outcomes (Project ECHO), a tele-education model developed at the University of New Mexico, utilizes videoconference technology to help PCPs gain the knowledge and self-efficacy to deliver complex specialty medical care. A group of interdisciplinary specialists located at a distant “hub” video links with community PCPs for regularly scheduled didactic sessions and case presentations. ECHO creates “knowledge networks” that promote learning and the rapid dissemination of current treatment research and epidemiological trends [80]. It has been proven effective for HCV treatment and has been applied to a range of other conditions [80–82]. An Integrated Addictions and Psychiatry Tele-ECHO clinic has recruited physicians to complete the 8-h buprenorphine training, thus allowing them to provide MAT. Since 2006, over 175 physicians in New Mexico have completed training through this program [80]. Tele-education may be particularly useful in rural or other underserved areas where access to specialty care or academic medical centers is limited.

#### mHealth Phone Apps

mHealth—the use of mobile apps and text messaging to promote health—has grown dramatically in the past decade. While mHealth approaches lack rigorous evaluation and are still early in development, mHealth holds the promise of providing cost-effective tools to manage care and promote health behaviors [83–85]. mHealth can provide a private communication platform that permits flexible reminders and content [86–88]. mHealth acceptability has been investigated among people who use drugs (PWUD) and are engaged in HIV or SUD treatment [88]. Results suggest that the majority of PWUD are interested in mHealth to promote health and adherence to HIV treatment. In the context of HIV, HCV, and OUD care, mHealth apps provide a platform to virtually integrate care by facilitating communication and the dissemination of information between patients and providers with potential to increase care coordination, both between care teams from different clinics and among team members at a clinic with co-located services. This approach, however, is limited by cost (usage of minutes) and lack of cell phone coverage in some rural areas.

A-CHESS is a smartphone app designed by researchers at the University of Wisconsin-Madison and is being evaluated for reducing illicit opioid use among individuals receiving MAT [79]. The app provides a platform for information sharing and SUD treatment support tools, such as cognitive behavioral therapy booster sessions; GPS-based location monitoring to predict locations that may place the individual at risk of drug use or unsafe sex; a “help” button that shows a list of preapproved supports including phone numbers; and distractive activities. For participants who are HIV and/or HCV positive, A-CHESS delivers health education content, and links to clinical care/case-management services. While still in development, it is a promising mHealth strategy to coordinate a patchwork of treatment services and support, especially in rural areas.

## Research Gaps and Future Directions

**Fig. 1:**
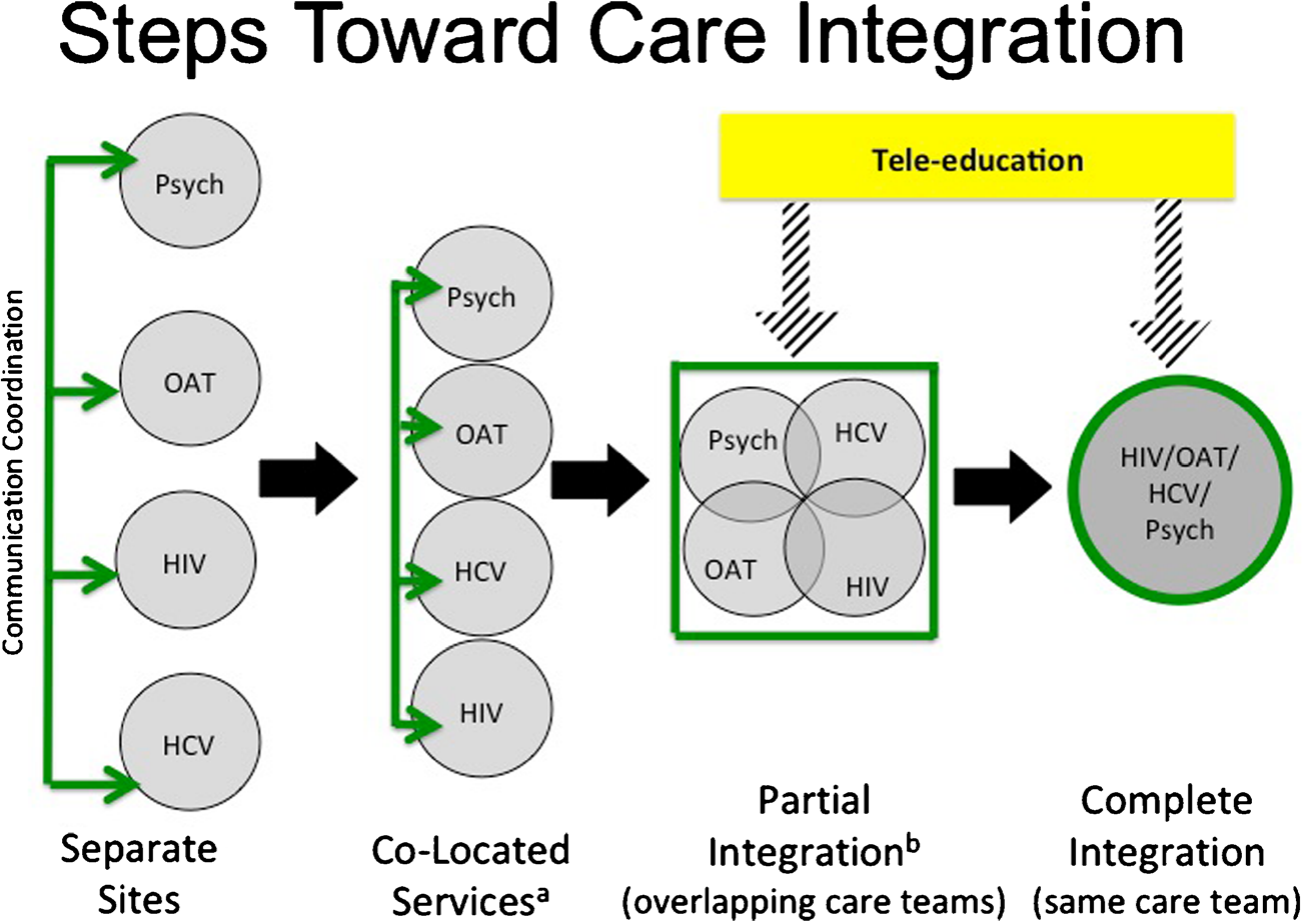
Steps toward HIV, opioid use disorder (OUD), and hepatitis C virus (HCV) care integration. ^a^Co-located services include models that provide two or more services at the same site. ^b^Partial integration includes models that provide two or more services with overlapping care teams

Our review identified several areas with limited information. The development of strategies to increase communication and teamwork across care teams may increase retention and success in care and may be more rewarding to both patients and practitioners. This is particularly important for the expansion of care in rural communities. Research is needed on optimal approaches to delivering harm reduction services in isolated rural communities, as well as strategies to effectively fold addiction, HIV, and HCV care into rural primary care practices that may already be overburdened and under-resourced. While tele-education has proven to be an exciting and effective strategy, additional research is needed to identify best practices and limitations, especially in areas without reliable internet access. Figure 1 depicts the potential pathway of integration from separate specialty clinics to a model of integrated care, supported by tele-education platforms.

Secondly, no studies have evaluated differing levels of engagement across racial, ethnic, and gender groups. Data are needed to better engage and care for diverse populations to prevent HIV and HCV among individuals with OUD. Work is especially needed to address the deleterious racial and ethnic legacies of the “War on Drugs”, as well as the pervasive stigma and distrust surrounding treatment—both for addiction and mental health care.

Third, research is needed to inform the delivery and integration or co-location of psychiatric care for a population with OUD and other co-morbidities. Information on how to best treat anxiety and depression among individuals with OUD and HIV is needed to inform treatment paradigms that do not exacerbate substance use, such as combined opioid and benzodiazepine dependence. Another significant barrier to the scale-up of integrated models of care is cost. To address this, future studies should consider including a budget impact analysis as part of the evaluation process. At a system-level approach, health insurance and payment reform are needed to allow reimbursement for care by mid-level practitioners, outreach workers, and peer navigators within integrated or co-located models of care, as well as telemedicine services.

Finally, increasing access to harm reduction services is essential to preventing HIV and HCV, including access to sterile injection equipment and a range of treatment options for OUD and HIV/HCV. Expansion of funding and political support is needed for developing innovative harm reduction programs, including pharmacy-based syringe exchange and increased provision of syringe prescriptions by clinicians.

## Limitations

Our review has several limitations. Due to the nature of a literature search, our description of care models is biased toward interventions connected with academic research institutions. While we included conference abstracts and searched the “gray literature” (materials and research produced by organization outside of traditional peer-reviewed academic publishing) for descriptions of care models not fully described in peer-reviewed articles, our search may not have captured all integrated or co-located treatment paradigms. Secondly, we restricted our search to models of care within the USA to provide examples of models most relevant to the current opioid epidemic. International programs, however, provide other examples of effective care models. Future research should look to incorporating aspects of successful international models to inform program development in the USA. Lastly, none of the models explicitly addressed provision of PrEP.

## Conclusion

The design and scale-up of integrated or co-located, multidisciplinary care models that engage and retain individuals in HIV, HCV, OUD, and mental health care are critical to preventing the continued spread of HIV and HCV in the context of the current opioid epidemic. Key components of such care models are the provision of (1) MAT to reduce the risk for HIV and HCV acquisition and to prevent HCV re-infection, (2) HIV and HCV treatment to prevent transmission through shared injection equipment and unsafe sex, (3) access to PrEP and to syringe services programs to prevent HIV and HCV, and (4) behavioral health services to address psychiatric co-morbidities. Communities in different locales may require different models. Examples of current models of care in five critical settings—primary care, HIV care, OPT/MAT, harm reduction programs, and transitional clinics—provide a good starting point for future innovation.

## Electronic Supplementary Material

ESM 1 (DOCX 67 kb)
