# Supplementary material for: Integrated Models of Care for Individuals with Opioid Use Disorder: How Do We Prevent HIV and HCV?
Source: Curr HIV/AIDS Rep. 2018 May 17;15(3):266–75. doi: 10.1007/s11904-018-0396-x (PMC6003996; doi:10.1007/s11904-018-0396-x)
Supplement: Supplementary file 1 — (DOCX 67 kb) [file 11904_2018_396_MOESM1_ESM.docx]

| **Table S. 1. Search Strategy** | | | | |
| --- | --- | --- | --- | --- |
|  | **Database** | | | |
| Concept | PsychInfo | EMBASE | Scopus | Ovid Medline |
| Medication-Assisted Treatment | ((Opioid*.tw.) OR (Opioid*.mp.)) OR exp Opiates/  AND  (Medication-assisted treatment.id.) OR (medication assisted adj2 (treatment* or therap*.mp.)) OR (maintenance therapy/) OR (buprenorphine/) OR (buprenorphine.mp.) OR (methadone maintenance/) OR (methadone therapy/) OR (methadone/) OR (naltrexone/) OR (methadone.mp.) OR (naltrexone.mp.) | ((Opioid*.tw.) OR (Opioid*.mp.)) OR Opiate/))  AND  (Medication-assisted treatment*.mp.) OR (medication assisted adj2 (treatment* or therap*.mp.)) OR  (methadone.mp.) OR (naltrexone.mp.) | (TITLE-ABS-KEY ("Opioid*") OR TITLE-ABS-KEY ("Opiate*") )  AND  ( TITLE-ABS-KEY ( "Medication-assisted treatment*" )  OR  TITLE-ABS-KEY ( "medication assisted adj2 (treatment* or therap*)" )  OR  TITLE-ABS-KEY ( "methadone" )  OR  TITLE-ABS-KEY ( "naltrexone" ) ) | Opioid*.tw. OR Opioid*.mp. OR exp Analgesics, Opioid/ OR exp opioid-related disorders/ OR exp opiate alkaloids/  AND  ((Medication-assisted treatment.id.) OR (medication assisted adj2 (treatment* or therap*.mp.)  OR  (methadone.mp.) OR (naltrexone.mp.)) |
| HIV | exp HIV/ OR HIV infection.mp. OR HIV-infected.mp. OR HIV-positive.mp OR HIV positive.mp | exp Human Immunodeficiency virus infection/ OR HIV infection.mp. OR HIV-infected.mp. OR HIV-positive.mp OR HIV positive.mp | (TITLE-ABS-KEY (“Human Immunodeficiency virus infection”) OR TITLE-ABS-KEY ("HIV infection" )  OR  TITLE-ABS-KEY ( "HIV-infected" )  OR  TITLE-ABS-KEY ( "HIV-positive" )  OR  TITLE-ABS-KEY ( "HIV positive" ) | exp HIV Infections/ OR HIV infection.mp. OR HIV-infected.mp. OR HIV-positive.mp OR HIV positive.mp |
| HCV | hepatitis c.tw. | exp Hepatitis C/ OR hepatitis c.tw. | TITLE-ABS-KEY (“hepatitis c”) | exp Hepatitis C/  OR hepatitis c.tw. |

APPENDIX

| **Table S.2. Models of Care** | | | | | | |
| --- | --- | --- | --- | --- | --- | --- |
| **Model of Care, Setting** | **Population(s) served** | **OUD Medications available** | **Mental Health care provided** | **HIV care provided** | **HCV care provided** | **Reference** |
| *HIV Care Sites* | | | | | | |
| HIV primary care clinic at Boston Medical Center (FAST PATH program) | Urban-located individuals with an OUD and who are HIV-positive | Buprenorphine | 1. Addiction counselor available  2. Motivational interviewing and CBT provided through group and individuals sessions | 1. A general internist primary care doctor who is experienced with HIV treatment provides HIV and primary care  2. The clinic is embedded within a hospital and has ready access to subspecialty care | Not described | (1,2) |
| Immunology Center at Miriam Hospital in Providence, Rhode Island* | Individuals who have an OUD and are HIV-positive | Buprenorphine | Not described | Physicians with specialty training in infectious disease provide care to patients | Patients who are coinfected with HIV/HIV receive care from the Coinfection Clinic within the Immunology Center | (3) |
| Nathan Smith HIV Clinic at Yale New Haven Hospital* | Individuals with an OUD and who are HIV-positive | Buprenorphine | Onsite psychiatric and social work services | Specialty HIV primary care center affiliated with Yale-New Haven Hospital | Onsite Hepatitis C testing and treatment | (4) |
| Pilot study at Ruth M. Rothstein CORE center in Chicago, IL* | Individuals with an OUD and who were HIV-positive | Participants in intervention arm of RCT were given a 380mg dose of extended release naltrexone (XR-NTX; Vivitrol**^®^**) at initiation, 4, 8, and 12 weeks | Mental health providers were available onsite to perform psychosocial evaluations, schedule outpatient therapy sessions, refer to psychiatric services located with the greater CORE center, and manage psychiatric medications  (Psychiatric services not part of study) | Specialty HIV care center affiliated with the Cook County Hospital | Hepatitis treatment was available onsite (not provided as part of study) | (5) |
| Randomized control trial with two HIV clinics in Rhode Island | Individuals with an OUD, engaged in care at a nearby methadone clinic and HIV-positive | Methadone | Therapist was available onsite  Suicidality was assessed weekly with the Brief Depression Index-II survey (BDI-II)  Psychiatrist was available onsite | HIV care was provided by physicians trained in infectious disease | HCV treatment was overseen weekly by HIV care team  HIV care team coordinated with methadone clinic to provided daily medications | (6) |
| *Primary Care Sites* | | | | | | |
| General internal medicine clinic at Boston Medical Center (FAST PATH program) | Urban-located individuals with an OUD and who are at high risk for HIV infection (IUD in previous 30 days or high-risky sexual activity in previous 6mo) | Buprenorphine | Addiction counselor available  Motivational interviewing and CBT provided through group and individuals sessions | Referred to HIV clinic at BMC | Not described | (1,2) |
| OBOT Clinic at within the adult primary care clinic at Boston Medical Center | Urban-located adults with an OUD | Buprenorphine | Patients with active psychiatric diagnosis are co-managed with a psychiatrist  Patients were referred to counseling services both onsite and offsite | Not described | All patients at intake are screened for HCV & HCV treatment provided onsite  The clinic uses a “collaborative care model” with nurse care managers, primary care physicians, and pharmacists working in teams to provide care and manage patients | (7,8) |
| Federally Qualified Health Clinic associated with Montefiore Medical Center* | Urban-located individuals | Buprenorphine | Limited psychiatric and mental health services available on site | Clinic received Ryan White CARE Act funding  Physicians with specialty training in HIV care available onsite | Full time care coordination services provided by New York City’s Check Hep C Patient Care Coordinator Program  Care provided by physician with specialty training in HCV and addiction medicine | (9–11) |
| *Specialty Opioid Treatment Program* | | | | | | |
| DoSA’s OTP Clinics:    *12 OTP clinics in the Bronx, New York operated by Albert Einstein School of Medicine’s Division of Substance Abuse (DoSA) and Montefiore Medical Center* | Urban-located individuals with an OUD | Methadone  Buprenorphine | Onsite psychiatry services are available | HIV testing, treatment, and counseling is available onsite. The clinic will help coordinate HIV care elsewhere if a patient prefers to receive treatment offsite.  The clinics have been the site of several pilot interventions (e.g STAR and STAR*DOT). These interventions have involved motivational interviewing and cognitive behavioral techniques to support treatment readiness and directly observed therapy. | HCV testing, treatment, and counseling is available onsite.  The clinic has developed a HCV support groups to provide onsite programs for patients to discuss and learn about, as well as to foster an advocacy community.  The clinic has developed a Peer Educators program, that employs HCV-positive “peers” to organize onsite and offsite activities, escort patients to liver biopsies, and provide logistical clinical support. | (10,12–21) |
| San Francisco General Hospital Opiate Treatment Program | Individuals with an OUD | Methadone | Onsite psychiatric treatment | HIV care is provided onsite. The program has been the site of ART adherence studies, including a contingency management voucher intervention with medication counseling & DAART. | Not described | (22–24) |
| Bay Area Addiction Research and Treatment (BAART), Market Street Clinic | Individuals with an OUD | Methadone | Not described | The clinic provides HIV care onsite. The clinic participated in a RCT studying a voucher-based incentive program to improve adherence to ART. | Not described | (22,23) |
| OTP clinics in Baltimore participating in a DAART intervention  (*Baltimore VA Drug Dependency Program; Program of Alcohol and other Drug Dependencies;* *Man Alive, Inc; New Hope Treatment Center; Day Break Methadone Clinic)* | Individuals with an OUD | Methadone  Buprenorphine | Not described | MAT and ART were packaged together and directly administered to individuals in a private location. Take home ART doses were provided for evenings, weekends, holidays, and weekdays when a participant did not attend the OTP. Participants also received three days’ worth of “emergency ART doses.” | Not described | (25–27) |
| The APT Foundation in New Haven, Connecticut | Individuals with an OUD | Methadone  Buprenorphine | Psychiatric services are available onsite  All patients beginning treatment undergo psychiatric evaluation by a physician with specialty training in psychiatry and/or addiction medicine | Opt-out HIV testing for new patients  Education, screening, and counseling provided onsite. Linkage to off-site treatment and care available. | Opt-out HCV testing for new patients  Education, evaluation and treatment provided onsite  Clinic staff, with patients’ permission, provide screening to contacts who may be been infected  Care provided by physicians with experience in HCV with coordination with social workers and psychiatrists | (28) |
| South Center Rehabilitation Center of Cornell Scott-Hill Health Center in New Haven, Connecticut | Individuals with an OUD | Methadone  Buprenorphine  Naltrexone | Licensed mental health providers are available onsite  The clinic integrates psychiatric care with substance abuse treatment | Opt-out HIV testing for new patients  Evaluation and treatment provided onsite  Care provided by physicians, nurses, counselors | Opt-out HCV testing for new patients  Evaluation and treatment provided onsite  Care provided by physicians, nurses, counselors | (29) |
| *Transitional Clinics* | | | | | | |
| Randomized control trial | Individuals transitioning from jail or prison who were HIV-positive with an OUD | Methadone  Buprenorphine (induction within 30 days post-release with day of release as optimal date) | Receipt of MAT was contingent on attendance at a weekly counseling session. | Participants were randomized to directly administered ART or treatment as usual. The DARRT participants were given ART daily with MAT and other chronic medications | Not described | (30,31) |
| Bronx Transitions Clinic (BTC) | Individuals who have been formerly incarcerated | Buprenorphine | Psychiatric services are provided onsite | Testing, treatment, and counseling are available onsite. Case management services are also available both through the BTC and a partner agency. | Testing, treatment, and counseling are available onsite. | (30,31) |
| *Community-Based Harm Reduction Programs* | | | | | | |
| Mobile Medical Clinic in New Haven, CT* | 1. Individuals in underserved city neighborhoods, typically without insurance and/or a traditional primary care provider  2. PWID | Buprenorphine  *Linkage to an OTP available | Psychiatric nurses and behavioral health counselors are available by appointment | 1. Testing, DAART, and case-management services provided onsite  2. Infectious disease specialist is available for consult  3. Linkage to primary care and HIV specialty clinics | 1. Testing and counseling services provided  2. Infectious disease specialist is available for consult  3. Linkage to care | (32,33) |
| *Emerging Models* | | | | | | |
| Telemedicine/ Tele-education (START) | Individuals who have an OUD and are HCV-positive and receiving methadone at an OTP | 1. Methadone | Not described | Not described | All individuals are provided an HCV evaluation via telemedicine. Laboratory tests are done onsite. A physician assistant (onsite) and a liver specialist (remote) review and enter patient data weekly into an EHR. Medications for HCV (DAAs) are administered with methadone. | (34–36) |
| Telemedicine/Tele-education (Project Echo) | Individuals with an OUD or HCV or HIV | Buprenorphine | Project ECHO utilizes video conference technology to help primary care physicians (PCPs) gain the knowledge and self-efficacy to deliver complex specialty medical care. The system involves having a group of inter-disciplinary specialists, who are located at a distant “hub” (The Integrated Addictions and Psychiatry (IAP) teleECHO clinic). The video link community PCPs for a series of weekly 2-hour training sessions and case presentations. The sessions create “knowledge networks” that promote learning and allow for the rapid dissemination of current research and epidemiological trends. Participation in the sessions is free of charge. The facilitators/specialists include an addiction specialist, a psychiatrist, a licensed clinical social worker with addiction expertise, a psychiatric nurse or psychiatric nurse specialist, and a community health worker. | | | (37–40) |
| mHealth (A-CHESS mobile app) | Individuals with an OUD  (The ongoing study of this intervention is recruiting individuals from outpatient detoxification and OTP programs) | Methadone  Buprenorphine  Naltrexone | A-CHESS’s platform provides several behavioral health interventions, including a “help” bottom that enables the user to contact pre-approved individuals for support; audio/video relaxation recordings; and cognitive behavior therapy booster sessions that correspond to in person CBT sessions | All users are asked about and prompted to seek HIV and HCV testing.  For HIV- and/or HCV-positive users, A-CHESS collects data on risk behaviors and provides behavior change interventions that are tailored to users’ self-reported readiness to change. Users are also delivered multimedia health education materials and location-specific links to clinical care and case management services. | | (41) |
| *** Clinic was a participating site in the HRSA-funded Special Project of National Significance Initiative: An Evaluation of Innovative Methods for Integrating Buprenorphine Opioid Abuse Treatment in HIV Primary Care Settings, 2004-2009 (BHIVES)** | | | | | | |

APPENDIX REFERENCES

1. Drainoni M-L, Farrell C, Sorensen-Alawad A, Palmisano JN, Chaisson C, Walley AY. Patient perspectives of an integrated program of medical care and substance use treatment. AIDS Patient Care STDs. 2014;28(2):71–81.

2. Walley AY, Palmisano J, Sorensen-Alawad A, Chaisson C, Raj A, Samet JH, et al. Engagement and Substance Dependence in a Primary Care-Based Addiction Treatment Program for People Infected with HIV and People at High-Risk for HIV Infection. J Subst Abuse Treat. 2015;59(kai, 8500909):59–66.

3. Taylor LE, Maynard MA, Friedmann PD, MacLeod CJ, Rich JD, Flanigan TP, et al. Buprenorphine for human immunodeficiency virus/hepatitis C virus-coinfected patients: Does it serve as a bridge to hepatitis C virus therapy? J Addict Med. 2012;6(3):179–85.

4. Tetrault JM, Moore BA, Barry DT, O’Connor PG, Schottenfeld R, Fiellin DA, et al. Brief versus extended counseling along with buprenorphine/naloxone for HIV-infected opioid dependent patients. J Subst Abuse Treat. 2012;43(4):433–9.

5. Korthuis PT, Lum PJ, Vergara-Rodriguez P, Ahamad K, Wood E, Kunkel LE, et al. Feasibility and safety of extended-release naltrexone treatment of opioid and alcohol use disorder in HIV clinics: A pilot/feasibility randomized trial. Addiction. 2017;112(6):1036–44.

6. Taylor LE, Bowman SE, Chapman S, Zaller N, Stein MD, Cioe PA, et al. Treatment for hepatitis C virus genotype 1 infection in HIV-infected individuals on methadone maintenance therapy. Drug Alcohol Depend. 2011;116(1–3):233–7.

7. Carey KJ, Huang W, Linas BP, Tsui JI. Hepatitis C virus testing and treatment among persons receiving buprenorphine in an office-based program for opioid use disorders. J Subst Abuse Treat. 2016;66:54–9.

8. Alford DP, LaBelle CT, Kretsch N, Bergeron A, Winter M, Botticelli M, et al. Collaborative care of opioid-addicted patients in primary care using buprenorphine: five-year experience. Arch Intern Med. 2011;171(5):425–31.

9. Cunningham C, Giovanniello A, Sacajiu G, Whitley S, Mund P, Beil R, et al. Buprenorphine Treatment in an Urban Community Health Center: What to Expect. Fam Med. 2008;40(7):500–6.

10. Cunningham CO, Sohler NL, Cooperman NA, Berg KM, Litwin AH, Arnsten JH. Strategies to improve access to and utilization of health care services and adherence to antiretroviral therapy among HIV-infected drug users. Subst Use Misuse. 2011;46(2–3):218–32.

11. Norton BL, Beitin A, Glenn M, DeLuca J, Litwin AH, Cunningham CO. Retention in buprenorphine treatment is associated with improved HCV care outcomes. J Subst Abuse Treat. 2017 Apr;75:38–42.

12. Batchelder AW, Peyser D, Nahvi S, Arnsten JH, Litwin AH. “Hepatitis C treatment turned me around:” Psychological and behavioral transformation related to hepatitis C treatment. Drug Alcohol Depend. 2015;153:66–71.

13. Berg KM, Mouriz J, Li X, Duggan E, Goldberg U, Arnsten JH. Rationale, design, and sample characteristics of a randomized controlled trial of directly observed antiretroviral therapy delivered in methadone clinics. Contemp Clin Trials. 2009;30(5):481–9.

14. Berg KM, Litwin A, Li X, Heo M, Arnsten JH. Directly observed antiretroviral therapy improves adherence and viral load in drug users attending methadone maintenance clinics: A randomized controlled trial. Drug Alcohol Depend. 2011;113(2–3):192–9.

15. Cooperman NA, Parsons JT, Chabon B, Berg KM, Arnsten JH. The development and feasibility of an intervention to improve HAART adherence among HIV-positive patients receiving primary care in methadone clinics. In: HIV Treatment Adherence: Challenges for Social Services [Internet]. 2013. p. 101–20. Available from: https://www.scopus.com/inward/record.uri?eid=2-s2.0-84905793579&doi=10.1300%2fJ187v06n01_07&partnerID=40&md5=803b9303a04ed01fc5cff207e938b5cc

16. Cooperman NA, Heo M, Berg KM, Li X, Litwin AH, Nahvi S, et al. Impact of adherence counseling dose on antiretroviral adherence and HIV viral load among HIV-infected methadone maintained drug users. AIDS Care. 2012;24(7):828–35.

17. Litwin AH, Harris Jr KA, Nahvi S, Zamor PJ, Soloway IJ, Tenore PL, et al. Successful treatment of chronic hepatitis C with pegylated interferon in combination with ribavirin in a methadone maintenance treatment program. J Subst Abuse Treat. 2009;37(1):32–40.

18. Litwin AH, Arnsten J, Heo M, Li X, Hidalgo J. Directly observed HCV treatment in methadone clinics-preliminary results. J Int Assoc Physicians AIDS Care. 2011;10(3):205.

19. Litwin AH, Berg KM, Li X, Hidalgo J, Arnsten JH. Rationale and design of a randomized controlled trial of directly observed hepatitis C treatment delivered in methadone clinics. BMC Infect Dis. 2011;11:315.

20. Roose RJ, Cockerham-Colas L, Soloway I, Batchelder A, Litwin AH. Reducing barriers to hepatitis C treatment among drug users: An integrated hepatitis C peer education and support program. J Health Care Poor Underserved. 2014;25(2):652–62.

21. Stein MR, Soloway IJ, Jefferson KS, Roose RJ, Arnsten JH, Litwin AH. Concurrent group treatment for hepatitis C: implementation and outcomes in a methadone maintenance treatment program. J Subst Abuse Treat. 2012;43(4):424–32.

22. Barnett PG, Sorensen JL, Wong W, Haug NA, Hall SM. Effect of incentives for medication adherence on health care use and costs in methadone patients with HIV. Drug Alcohol Depend. 2009;100(1–2):115–21.

23. Sorensen JL, Haug NA, Delucchi KL, Gruber V, Kletter E, Batki SL, et al. Voucher reinforcement improves medication adherence in HIV-positive methadone patients: a randomized trial. Drug Alcohol Depend. 2007;88(1):54–63.

24. Sorensen JL, Haug NA, Larios S, Gruber VA, Tulsky J, Powelson E, et al. Directly administered antiretroviral therapy: pilot study of a structural intervention in methadone maintenance. J Subst Abuse Treat. 2012;43(4):418–23.

25. Lucas GM, Mullen BA, Galai N, Moore RD, Cook K, McCaul ME, et al. Directly Administered Antiretroviral Therapy for HIV-Infected Individuals in Opioid Treatment Programs: Results from a Randomized Clinical Trial. PLoS ONE. 2013;8(7):e68286.

26. Mullen BA, Cook K, Moore RD, Rand C, Galai N, McCaul ME, et al. Study design and participant characteristics of a randomized controlled trial of directly administered antiretroviral therapy in opioid treatment programs. BMC Infect Dis. 2011;11:45.

27. Lucas GM, Mullen BA, McCaul ME, Weidle PJ, Hader S, Moore RD. Adherence, drug use, and treatment failure in a methadone-clinic-based program of directly administered antiretroviral therapy. AIDS Patient Care STDs. 2007;21(8):564–74.

28. Butner JL, Gupta N, Fabian C, Henry S, Shi JM, Tetrault JM. Onsite treatment of HCV infection with direct acting antivirals within an opioid treatment program. J Subst Abuse Treat. 2017 Apr;75:49–53.

29. Bruce RD, Eiserman J, Acosta A, Gote C, Lim JK, Altice FL. Developing a modified directly observed therapy intervention for Hepatitis C treatment in a methadone maintenance program: Implications for program replication. Am J Drug Alcohol Abuse. 2012;38(3):206–12.

30. Springer SA, Qiu J, Saber-Tehrani AS, Altice FL. Retention on buprenorphine is associated with high levels of maximal viral suppression among HIV-infected opioid dependent released prisoners. PLoS ONE [Internet]. 2012;7(5). Available from: http://ovidsp.ovid.com/ovidweb.cgi?T=JS&PAGE=reference&D=psyc9&NEWS=N&AN=2012-17123-001 http://journals.plos.org/plosone/article/file?id=10.1371/journal.pone.0038335&type=printable http://journals.plos.org/plosone/article/file?id=10.1371/journal.pone.0038335&type=printable

31. Springer SA, Chen S, Altice FL. Improved HIV and substance abuse treatment outcomes for released HIV-infected prisoners: the impact of buprenorphine treatment. J Urban Health Bull N Y Acad Med. 2010;87(4):592–602.

32. Sylla L, Bruce RD, Kamarulzaman A, Altice FL. Integration and co-location of HIV/AIDS, tuberculosis and drug treatment services. Int J Drug Policy. 2007;18(4):306–12.

33. Gibson BA, Morano JP, Walton MR, Marcus R, Zelenev A, Bruce RD, et al. Innovative Program Delivery and Determinants of Frequent Visitation to a Mobile Medical Clinic in an Urban Setting. J Health Care Poor Underserved. 2017;28(2):643–62.

34. Talal A, Andrews P, McLeod A, Zeremski M, Chen Y, Sylvester C, et al. Integrated, co-located, telemedicine-based treatment approaches for hepatitis C virus (HCV) management for individuals on opioid agonist treatment. J Hepatol. 2016;64(2 SUPPL. 1):S747.

35. Brown S-E, Vagenas P, Konda KA, Clark JL, Lama JR, Gonzales P, et al. Men Who Have Sex with Men in Peru: Acceptability of Medication-Assisted Therapy for Treating Alcohol Use Disorders. Am J Mens Health [Internet]. 2015 Mar 17 [cited 2017 Mar 13]; Available from: http://www.ncbi.nlm.nih.gov/pmc/articles/PMC4573823/

36. Brown JL, Gause NK, Lewis D, Winhusen T. Examination of the Hepatitis C Virus care continuum among individuals with an opioid use disorder in substance use treatment. J Subst Abuse Treat. 2017 May;76:77–80.

37. Arora S, Kalishman S, Thornton K, Dion D, Murata G, Deming P, et al. Expanding access to hepatitis C virus treatment--Extension for Community Healthcare Outcomes (ECHO) project: disruptive innovation in specialty care. Hepatol Baltim Md. 2010 Sep;52(3):1124–33.

38. Komaromy M, Duhigg D, Metcalf A, Carlson C, Kalishman S, Hayes L, et al. Project ECHO (Extension for Community Healthcare Outcomes): A new model for educating primary care providers about treatment of substance use disorders. Subst Abuse. 2016 Jan 2;37(1):20–4.

39. Chan PPY, Mohsen W, Whelan M, Glass A, Ladera A, Mouton M, et al. Project ECHO: A novel tele-mentoring service to aid hepatitis C treatment in difficult-to-access populations. J Gastroenterol Hepatol Aust. 2017;32:67.

40. Arora S, Thornton K, Murata G, Deming P, Kalishman S, Dion D, et al. Outcomes of treatment for hepatitis C virus infection by primary care providers. N Engl J Med. 2011 Jun 9;364(23):2199–207.

41. Gustafson DH, Landucci G, McTavish F, Kornfield R, Johnson RA, Mares M-L, et al. The effect of bundling medication-assisted treatment for opioid addiction with mHealth: study protocol for a randomized clinical trial. Trials. 2016 Dec 12;17(1):592.
